# Development of the Japanese Version of Rushton Moral Resilience Scale (RMRS) for Healthcare Professionals: Assessing Reliability and Validity

**DOI:** 10.1155/2024/7683163

**Published:** 2024-11-05

**Authors:** Keiko Wataya, Masana Ujihara, Yoshitaka Kawashima, Shinichiro Sasahara, Sho Takahashi, Asako Matsuura, Adam Lebowitz, Hirokazu Tachikawa

**Affiliations:** ^1^Graduate School of Comprehensive Human Sciences, University of Tsukuba, Tsukuba, Japan; ^2^Department of Nursing, University of Tsukuba Hospital, Tsukuba, Japan; ^3^College of Nursing and Nutrition, Shukutoku University, Chiba, Japan; ^4^School of Arts and Letters, Meiji University, Chiyoda, Japan; ^5^Occupational and Aerospace Psychiatry Group, Institute of Medicine, University of Tsukuba, Tsukuba, Japan; ^6^Department of Disaster and Community Psychiatry, Institute of Medicine, University of Tsukuba, Tsukuba, Japan; ^7^Faculty of Health Science, Toho University, Funabashi, Japan; ^8^Department of General Education, Jichi Medical University, Shimotsuke, Japan

**Keywords:** mental health, moral distress, moral resilience, nurse, scale development

## Abstract

**Aim:** To translate the Rushton Moral Resilience Scale (RMRS) into Japanese and validate its applicability among Japanese healthcare professionals.

**Background:** To overcome daily challenges in the field of healthcare, in which moral difficulties are routinely encountered, the development of intervention methods to address moral suffering and moral distress is crucial.

**Methods:** We conducted a cross-sectional survey using a web-based questionnaire. The RMRS-16 was translated into Japanese and confirmed through back-translation. Reliability analyses (Cronbach's alpha and intraclass correlation coefficient [ICC]), confirmatory factor analyses (CFAs), correlation analyses, *t*-tests, and analysis of variance (ANOVA) were used to assess the validity of the scale.

**Results:** Participants comprised 1295 healthcare professionals, including 498 nurses. All subscales and the total scale had acceptable reliability values (α ≥ 0.70). CFA supported the original four-factor structure (response to moral adversity, personal integrity, relational integrity, and moral efficacy), with acceptable fit indices. The ANOVA results suggested that, among Japanese healthcare professionals, nurses and individuals from other professions showed lower average moral resilience scores compared to physicians, consistent with previous research on mental health and moral distress. In addition, women scored lower for moral resilience than men. However, the ICC values for the subscales of the RMRS were below acceptable levels, and the results of the standardized residual covariances also suggested a model misfit.

**Conclusion and Implications:** The reliability, validity, and utility of the Japanese version of the RMRS were generally supported. However, there were areas at the item level that required structural examination. The current findings suggest that there are cultural differences in the concept of moral resilience. Therefore, for future cultural comparisons, the original four-factor structure was maintained in the Japanese version without modifications. Further conceptual development of moral resilience is needed in Japanese healthcare.

## 1. Introduction

Moral distress is frequently experienced among nurses in healthcare settings, particularly in situations that involve numerous ethical challenges such as decision-making with patients and their families, managing patients with symptoms that are difficult to control, and in scenarios where prioritizing the quality of life for patients is not possible [1, 2]. Moral distress has been characterized as a negative emotion associated with a moral event, encompassing various types of moral tension, moral conflict, moral dilemma, moral uncertainty, and moral constraint [3]. Furthermore, moral distress has been reported to be associated with compassion fatigue, burnout syndrome, and secondary traumatic stress syndrome in the field of nursing [4]. Moral injury is described as a deep sense of transgression, including feelings of shame, grief, meaninglessness, and remorse resulting from having violated core moral beliefs [5]. Moral injury is considered to be the most severe form of moral distress, exhibiting positive correlations with depression, anxiety, burnout syndrome, and significant impairment of social functioning [1, 5, 6].

During the coronavirus disease 2019 (COVID-19) pandemic, deterioration of mental health caused by moral distress and moral injury was reported among healthcare professionals [6–8]. Specifically, among nursing staff, the intention to resign and burnout were reported to be associated with moral distress [9, 10]. In a previous survey conducted during the COVID-19 pandemic, nurses were found to experience moral distress resulting from working with limited resources and unclear communication about the safety of patients, visitors, and healthcare workers within their organization [11, 12]. Not only nurses but also other healthcare professionals reported an increase in moral issues, including moral injury, during the COVID-19 pandemic compared with the prepandemic period [13]. The prevalence of moral injury was reported to be 41.3% (95% confidence interval [CI]: 39.3%–43.0%) in 2020 among healthcare professionals [6].

One measure used to assess moral distress is the Moral Distress Scale (MDS) developed by Corley and colleagues in 2001 [14]. Subsequently, the Measure of Moral Distress for Healthcare Professionals (MMD-HP) was developed by Epstein and colleagues in 2019 [15]. These instruments have been used within intervention programs that aim to improve moral distress [16–19].

Existing intervention programs have conducted case reflection, educational sessions, and articulation of moral distress, but the reported effectiveness of addressing moral distress has not been consistent [16, 20]. However, improvements in moral confidence, ethical competence, and moral resilience have been reported in response to these interventions for ethical challenges [19, 21]. To overcome daily challenges in the field of healthcare, in which moral difficulties are routinely encountered, it is crucial to explore methods not only for improving individuals' moral distress but also for transforming inherent moral stress and suffering that cannot be eliminated. In addition, it is important to identify ways to enhance systems and methods for addressing moral distress.

Against this background, the concept of moral resilience among healthcare professionals has been proposed [22]. The concept of moral resilience for healthcare professionals comprises six pillars: (1) personal integrity, (2) relational integrity, (3) buoyancy, (4) self-regulation, (5) self-stewardship, and (6) moral efficacy [1, 23, 24]. The Rushton Moral Resilience Scale (RMRS) was developed to measure individuals' moral resilience, comprising four components: (1) response to moral adversity, (2) personal integrity, (3) relational integrity, and (4) moral efficacy. Each factor on the scale is defined as follows: response to moral adversity refers to the ability to face moral challenges through self-regulation, self-management, and moral efficacy. Personal integrity is the capacity to align one's values with actions to maintain moral wholeness in difficult situations. Relational integrity involves balancing one's own beliefs with openness and flexibility toward the beliefs and values of others. Moral efficacy reflects confidence in the face of resistance and the ability to resolve moral conflicts in ways that align with one's values [1, 24, 25]. A medium effect size negative correlation has been reported between buoyancy in moral resilience and both “depersonalization” and “emotional exhaustion” [26].

The development of intervention methods to address moral suffering and moral distress is crucial. In Japan, there is a need to explore new intervention methods to protect the moral well-being of healthcare professionals, and a need to establish indicators to measure the effectiveness of interventions. Therefore, the current study sought to validate and assess the reliability of the RMRS for use in Japanese populations.

## 2. Materials and Methods

### 2.1. Procedure

#### 2.1.1. Translation and Pilot Testing of the RMRS

With permission from the original developers, a Japanese version of the RMRS questionnaire was created. The translation process involved two researchers validating the appropriateness of the translation for each item. Subsequently, adjustments to the Japanese version were made through discussions with a third native Japanese translator. The adjusted translation, unfamiliar to the original text, underwent back-translation by a native English speaker with proficiency in medical knowledge. The back-translated version was compared with the original version to verify consistency. Adjustments to problematic items were made in Japanese. Clinical experts, including two psychiatrists, two occupational health physicians, two nurses, and one psychologist, then reviewed the translation. After obtaining linguistic consensus, we conducted a pilot test with a total of 14 participants aged 20–60, including two physicians, nine nurses, two public health nurses, and one midwife, and confirmed that there were no invalid responses.

#### 2.1.2. Scale Validation

**Figure 1:**
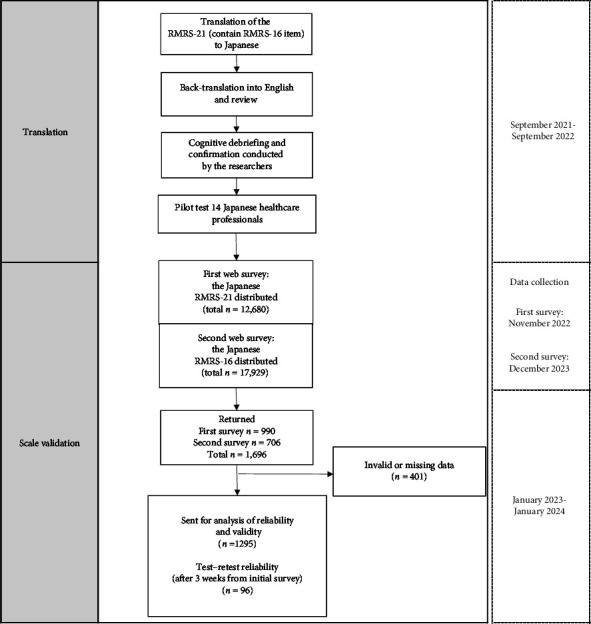
Study flow.

The research employed a web survey through a commissioned contract with the survey company Rakuten Insight (https://insight.rakuten.co.jp/), using a questionnaire created by the researchers, with data collection entrusted to Rakuten Insight up to the aggregation stage. The target population consisted of active healthcare professionals in Japan, aged 20–70, registered with Rakuten Insight, and during the survey period, surveys were distributed to 12,680–17,929 individuals who were registered. The surveyed professionals included physicians, nurses, midwives, pharmacists, physiotherapists, occupational therapists, speech therapists, social workers, mental health and welfare workers, laboratory technicians, and radiology technicians and were recruited from various healthcare organizations. This survey was conducted as a web-based survey distributed to healthcare professionals nationwide. As the affiliations of the respondents were not disclosed, the researchers checked all responses to determine if they were from healthcare professionals with the specified qualifications. Responses that did not meet this criterion were excluded from the analysis. To secure the sample size required for the analysis, the survey was conducted twice, first in November 2021, and then again in December 2022, maximizing the number of responses. To examine test–retest reliability, a follow-up RMRS survey was conducted with the initial respondents 3 weeks after their first response (Figure 1).

### 2.2. Sample Size and Sampling

We used the sample size calculation app (G^∗^ Power 3.1.9.7) to estimate the approximate sample size. Sample size calculation was set for *χ*^2^ test analysis, and statistical testing was conducted using a goodness-of-fit test. With the effect size set to 0.3, α error set to 0.05, *β* error set to 0.95, and degrees of freedom set to 98, the results yielded a required total sample size of 627. This study aimed to elucidate the structure of the scale, and because of the nature of the web survey, in which it was not possible to confirm respondents' interests and backgrounds, it was necessary to anticipate a large number of invalid responses [27]. Therefore, a minimum sample size of 1000 was set.

### 2.3. Missing Data

We excluded a total of 421 respondents, comprising those who did not hold qualifications in medical professions and those who answered all items on the questionnaire with the same number. Regarding the Kessler Psychological Distress Scale (K6), given the absence of reverse-scoring items and the focus of the K6 on measuring psychiatric symptoms, respondents who answered all questions with the same number were included in the analysis. Finally, we included 1295 respondents who provided appropriate answers for all items on the RMRS in the analysis.

### 2.4. Measures

#### 2.4.1. RMRS

The original version of the RMRS was developed in English by researchers in the United States [1, 25]. The original version consists of 16 items, comprising four subscales: response to moral adversity (e.g., difficult ethical situations leave me feeling powerless [reverse-scored]), personal integrity (e.g., I have the conviction to act in accordance with my values), relational integrity (e.g., my fear can cause me to act in a way that compromises my values [reverse-scored]), and moral efficacy (e.g., when I am confronted with an ethical challenge, I am able to articulate the ethical conflict). In the most recent report by the original authors [1], an examination of the items related to “personal integrity” and a reconsideration of the number of items for each subscale were conducted. The current 16-item scale has been reported to demonstrate good internal consistency, validity, and reliability. The RMRS comprises 16 items with responses on a four-point Likert scale: 1 (*disagree*), 2 (*somewhat disagree*), 3 (*somewhat agree*), and 4 (*agree*). Scores on the RMRS-16 and its subscales range from 1 to 4, with higher scores indicating greater moral resilience. Cronbach's alpha for the referenced validation study by Rushton et al. [1] was 0.85 in the full sample.

### 2.5. Other Measures

#### 2.5.1. The Japanese Version of the MMD-HP

We used the Japanese version of the MMD-HP [28]. The MMD-HP is a 27-item scale with a four-point Likert scale (0–4) for measuring the frequency and intensity of moral distress. The overall MMD-HP score is derived by summing the item composite scores (range: 0–432), with higher scores indicating elevated levels of moral distress. Cronbach's alpha coefficient of the scale has been confirmed as 0.91.

#### 2.5.2. The K6

Depression and anxiety were measured using the K6 [29]. The K6 is a six-item scale using a five-point Likert scale (ranging from 0 to 4). Total scores for the six items range from 0 to 24 and have been used as an indicator of severe mental disorders. The Japanese version of the K6 has also been standardized [30].

#### 2.5.3. The Japanese Version of the Maslach Burnout Inventory-General Survey (MBI-GS)

Burnout was measured using the Japanese version of the MBI-GS, developed by Kitaoka‐Higashiguchi et al. [31]. This version comprises three subscales: emotional exhaustion, depersonalization, and personal accomplishment, with a total of 16 items rated on a seven-point Likert scale (ranging from 0 to 6). Cronbach's alpha of the subscales for the referenced validation study by Kitaoka et al. was above 0.80.

#### 2.5.4. The Japanese Version of the Resilience Scale and Short Version of the RS (RS-14)

Resilience was assessed using the RS-14. The Japanese version of the RS-14, developed by Nishi in 2010, is a modification of the original Resilience Scale [32]. This scale measures 14 items on a seven-point Likert scale, with higher total scores indicating higher levels of resilience. Subsequent reports suggested the utility of this scale in a broader population [33]. Cronbach's alpha coefficient of the scale has been confirmed as 0.88.

#### 2.5.5. The Japanese Version of the Moral Efficacy Scale (J-MES)

The J-MES was developed by Japanese researchers [34] on the basis of a scale originally developed by May [35]. The scale measures the ethical efficacy of hospital nurses using a six-point Likert scale consisting of nine items. Cronbach's alpha for the referenced validation study by Inagaki et al. was 0.92.

### 2.6. Analyses

We used SPSS Statistics Version 28.0 (IBM, Tokyo, Japan) for Windows and SPSS AMOS Version 28.0 (IBM, Tokyo, Japan) to conduct the statistical analyses described below. A two-tailed *p* value of < 0.05 was considered to be statistically significant. We calculated and described the participants' characteristics as means, standard deviations (SDs), proportions, and frequencies.

Next, on the basis of previous research, we hypothesized that moral resilience scores would be associated with occupation, position, and educational background. This hypothesis was tested using one-way analysis of variance (ANOVA).

To test the reliability of the scale, we calculated Cronbach's alpha for the entire scale as well as for each factor to assess internal consistency. A minimum value of 0.70 is generally considered desirable [36]. In addition, we calculated the intraclass correlation coefficient (ICC) to assess test–retest reliability, using a two-way mixed effects model with absolute agreement. On the basis of the 95% CI of the ICC estimate, values less than 0.50 indicate poor reliability, values between 0.5 and 0.75 indicate moderate reliability, values between 0.75 and 0.9 indicate good reliability, and values greater than 0.90 indicate excellent reliability [37]. To test the validity of the scale, we examined concurrent validity by calculating Pearson's correlation coefficients using MMD-HP, K6, MBI-GS, RS-14, and J-MES. Furthermore, to assess the construct validity and cross-cultural validity, we conducted a confirmatory factor analysis (CFA) for 16 items, verifying the internal structure validated with the RMRS-16 in the original study. A nonsignificant *χ*^2^ test statistic suggests an acceptable fit between the model and the data. However, it is not advisable to rely solely on the *χ*^2^ test statistic to determine model fit, because it is highly sensitive to sample size and is often significant with large sample sizes, even when the model fits well [38]. It is considered difficult to designate specific cutoff values for each fit index. However, Hu and Bentler recommend using cutoff values close to 0.95 for comparative fit index (CFI), close to 0.08 for standardized root mean square residual (SRMR), and close to 0.06 for root mean square error of approximation (RMSEA) as criteria indicating a relatively good fit between the hypothesized model and observed data [39]. We proceeded with our analysis on the basis of these criteria.

For international comparisons, the mean differences between the Japanese population and the Canadian population on the RMRS were analyzed using a Student's *t*-test with summary statistics.

### 2.7. Ethical Considerations

We followed the guidelines of the Declaration of Helsinki in conducting this study [40]. The Ethics Committee of Tsukuba University approved this study (Approval no. 1818-1). In accord with the Ethical Guidelines for Life Science and Medical Research Involving Human Subjects, an explanation was given to subjects at the beginning of the web questionnaire form regarding the purpose of the survey. Participants were then asked to confirm their consent by checking a box on the web page to confirm that they understood the information provided and agreed to participate in the survey.

## 3. Results

### 3.1. Translation

The following points were discussed through the review of the back-translation and cognitive debriefing conducted by practicing clinicians. In the Japanese RMRS, the word “moral” in English was translated to “(moral),” and the word “ethics” was translated to “(rinri).” As a distinction, while “moral” is interpreted as subjective principles, “ethics” is considered to refer to objective principles within a specific group. The Japanese word “(doutoku)” encompasses the meanings of both the rightful principles of humans and the cultivation of good habits in humans. In medical settings, the term “ethics” is often used to denote objective standards, commonly translated as “ethics” in Japanese, which is also the case in the original RMRS, in which “ethics” is used to describe behavioral principles in medical contexts. In our research group, we believe that the concept of “moral resilience” encompasses not only societal norms but also personal beliefs and values. Therefore, we considered that it was appropriate to translate “moral” to “moral” rather than “ethics” or “morality.” We reported the content of the translation to the original developer and obtained their consent. As the final step of the translation process, a pilot test of the provisional version was conducted with 14 healthcare practitioners, and there were no unanswered items.

### 3.2. Demographic Characteristics

Of the respondents, 401 were excluded, comprising 296 respondents who answered all items with the same number on the survey scale and 105 respondents who were healthcare professionals without medical qualifications, such as administrative staff or care assistants. Thus, 1295 participants (76%) were included in the analysis.

The response rate was 39.7% for physicians, 38.5% for nurses, and 21.8% for other healthcare workers. Participants' mean age was 46.4 (SD: 12.4), and the number of years of experience in the current certification was 21.1 (SD: 12.3). The gender distribution in the sample was 51.2% male and 48.8% female. The primary practice locations were inpatient care settings (45.4%) and outpatient care settings (34.1%). More than 70% of participants were in staff employment roles. Regarding educational background, 64.5% of participants had graduated from universities and 34.1% had graduated from vocational colleges (Table 1).

### 3.3. Internal Consistency

Cronbach's alpha value for the reliability of the Japanese RMRS total scale was 0.85 (95% CI: 0.84–0.86). There were four items in each of the four factors (response to moral adversity (α = 0.79, 95% CI: 0.77–0.80), personal integrity (α = 0.75, 95% CI: 0.73–0.77), relational integrity (α = 0.79, 95% CI: 0.77–0.81), and moral efficacy (α = 0.71, 95% CI: 0.68–0.73)).

The ICC for the Japanese RMRS total scale in the test–retest analysis was 0.70 (95% CI: 0.58–0.79, *p* < 0.001), demonstrating low reliability. Among the factors, response to moral adversity was 0.63 (95% CI: 0.50–0.74, *p* < 0.001), personal integrity was 0.48 (95% CI: 0.31–0.62, *p* < 0.001), relational integrity was 0.45 (95% CI: 0.27–0.59, *p* < 0.001), and moral efficacy was 0.53 (95% CI: 0.37–0.66, *p* < 0.001) (Table 2).

### 3.4. CFA

**Figure 2:**
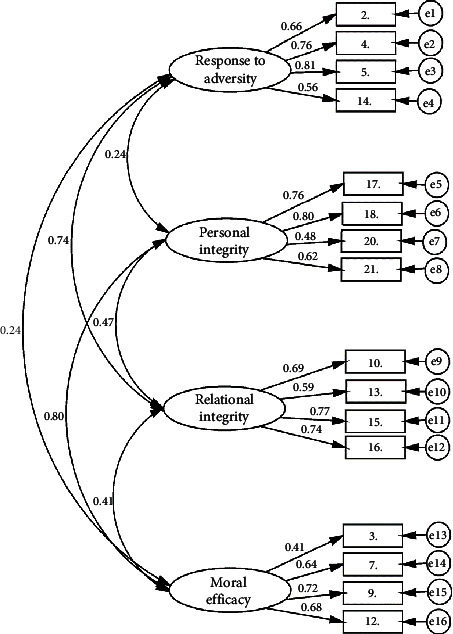
Results of confirmatory factor analysis.

We conducted a CFA to validate the four-factor structure of the RMRS original version (Figure 2). The fit indices are presented in Table 3, where the chi-square value of the model was *χ*^2^ 697.311 (98), *p*  <  0.001, and *χ*^2^ degrees of freedom value of the model was 7.115. The GFI was 0.935, the CFI was 0.917, and the adjusted goodness-of-fit index was 0.909. The SRMR was 0.055. The RMSEA of the model was 0.069 (95% CI: 0.064–0.074). The standardized factor loadings for the factors ranged from 0.410 to 0.814 and were all statistically significant at the *p* < 0.001 level.

### 3.5. Standardized Residual Covariance Analysis

The standardized residual covariance analysis revealed large negative residuals between Item 3 and Items 2 (−4.94), 4 (−5.82), 13 (−3.93), 15 (−5.85), and 16 (−4.18), as well as large positive residuals between Item 3 and Item 20 (+3.19). In addition, large negative residuals were observed between Item 15 and Item 18 (−3.51), whereas large positive residuals were found between Item 15 and Item 14 (+5.85).

### 3.6. Criterion Validity Correlations

The means, SD, and correlations of the measures are presented in Table 4. All measures exhibited significant correlations with each other. The overall RMRS showed significant negative associations with the K6 total score (*r* [648] = −0.41, *p*  < 0.001), MBI-GS total score (*r* [648] = −0.55, *p*  < 0.001), and MMD-HP total score (*r* [648] = −0.27, *p*  < 0.001). In addition, the RMRS demonstrated significant positive associations with the J-MES (*r* [648] = 0.57, *p*  < 0.001) and RS-14 (*r* [648] = 0.57, *p* < 0.001). Moreover, significant correlations were observed between the RMRS subscales and each psychological measure (Table 4).

### 3.7. RMRS Score

As shown in Table 4, the mean overall RMRS score was 2.66 (SD 0.41) for all participants. Positive correlations were observed among the factors, all of which were significant at the *p*  < 0.05 level. The comparison of the RMRS scores between the Japanese version of the RMRS in the current study and the RMRS original study [1] in Table 5 shows that Japanese healthcare professionals had significantly lower scores in both total scores and each factor score compared with Canadian healthcare professionals.

The differences in RMRS score by participant demographics are presented in Table 6. Two-tailed *t*-tests revealed that men had significantly higher RMRS total scores and all subscale scores (*t* [1293] = 11.62, *p* < 0.01; *t* [1293] = 9.30, *p* < 0.01; *t*[1293] = 7.12, *p* < 0.01; *t* [1293] = 10.19, *p* < 0.01; *t*[1293] = 5.30, *p* <  0.01) compared with women. One-way ANOVA with Tukey's follow-up test was used to examine differences in RMRS scores by professional background. Differences by professional role, employment role, primary practice location, and educational background were found in RMRS total scores (*F* [5, 1289] = 31.41, *p* < 0.00; *F* [2, 1292] = 29.98, *p* < 0.00; *F* [5, 1289] = 6.80, *p* < 0.00; *F* [3, 1283] = 29.13, *p* < 0.00). Multiple comparisons using Tukey's honestly significant difference test indicated that the total RMRS score for physicians and dentists was higher than that for nurses and other health professions, excluding social workers. In addition, individuals in staff positions had lower total RMRS scores and lower scores on all subscales compared to individuals in managerial positions. The RMRS total score and all subscales of healthcare professionals in the outpatient department had higher average scores compared with those in other departments. Furthermore, a significant difference was found between education levels, with higher levels of education being associated with higher scores, particularly in response to moral adversity.

## 4. Discussion

### 4.1. Summary of Results

The objective of the current study was to translate the RMRS into Japanese and validate its applicability for assessing Japanese healthcare professionals. We analyzed responses from 1295 participants, including nurses, physicians, and other qualified medical staff.

The internal consistency of the Japanese RMRS was found to be acceptable, with a Cronbach's alpha coefficient of 0.85. The test–retest reliability of the scale indicated poor to acceptable reliability with an ICC of 0.58–0.79. CFA was conducted on the basis of the original four-factor structure, and acceptable fit index values were obtained. The fit index results were generally consistent with those of previous studies, suggesting the structural validity of the Japanese version of the RMRS. However, the analysis of standardized residual covariances revealed that some items were significantly over or underestimated, indicating that further research is necessary to fully establish the structural validity of the Japanese version.

The results of the test–retest reliability, the analysis of standardized residual covariances, and the criterion-related validity assessment suggested that the concept of moral resilience was not clear among healthcare professionals in Japan. However, the results indicated that, compared with physicians, nurses and other medical professionals had lower scores for moral resilience. This reflects the situation in Japan's healthcare system and is consistent with previous reports on the mental health status of healthcare professionals in Japan [41–43]. Therefore, the findings suggested that RMRS scores may serve as an indicator of mental health status among healthcare professionals in Japan.

### 4.2. Interpretation of the Results

The internal consistency of the Japanese RMRS was found to be acceptable, with a Cronbach's alpha coefficient of 0.85. In addition, for the subscales, the Cronbach's alpha coefficient exceeded the acceptable value of 0.70. The ICC for the total RMRS in the test–retest was 0.70, indicating moderate reliability. However, the ICC for the subscales in our study was lower than those reported in a previous study [44], suggesting lower test–retest reliability, particularly for “personal integrity” and “relational integrity,” in the Japanese version of the RMRS. Two possible explanations for the results are as follows: first, the translation may have been difficult to comprehend; second, Japanese healthcare workers' understanding of morals and ethics may lack clarity.

CFA was conducted on the basis of the original four-factor structure and acceptable fit indices were obtained. Although the *χ*^2^ test statistic was significant, we chose to assess the model fit using other indicators because of the relatively large sample size. The fit indices were generally consistent with those of previous studies [1] However, some localized misfit was identified in the standardized residual covariances. The standardized residual covariance between Item 3 and Items 2, 4, 13, 15, and 16, as well as that between Items 15 and 18, exceeded the threshold of 2.58, indicating that the model overestimated the covariance between these items. In contrast, the standardized residual between Item 3 and Item 20, as well as that between Item 15 and Item 14, indicates potential underestimation of the factor structure. Item three of the moral efficacy subscale “I voice my ethical concerns in a way that others take seriously,” may not have effectively captured the ability and sense of efficacy it was intended to measure, potentially leading to difficulty for respondents in interpreting the item. Similarly, Item 15 of the relational integrity subscale, “I tend to be distracted by others' strong emotions when ethical conflicts occur,” may not have fully reflected the core concept of relational integrity, which involves the ability to reconcile one's own values with those of others. This disconnect may have caused respondents to perceive the item as conceptually distinct from the others. Therefore, further investigation is needed regarding Items 3 and 15, which appeared to contribute to both the underestimation and overestimation of the model.

The results of criterion validity correlation analysis revealed strong positive correlations between the total score and subcategories of RMRS, consistent with the original findings. Correlations of 0.5 or higher were observed between each scale and the total score, as well as the subscale scores of the RMRS, particularly with the MBI, J-MES, and RS-14. These results are in accord with previous reports, suggesting that the criterion-related validity was met [25].

These results indicate that, because of concerns about test–retest reliability and the fit of the scale at the item level, further re-evaluation of the scale's validity is necessary, even though the reliability and validity measures were generally acceptable.

Finally, we examined differences between respondents' characteristics and RMRS scores to assess the usefulness of the scale among Japanese healthcare professionals and to assess the utility of the scale among Japanese healthcare workers. Resilience is commonly perceived as the ability to cope with stress in challenging situations [32]. Therefore, the concept of resilience is considered to be strongly related to stress [24]. The current results revealed that nurses exhibited lower levels of moral resilience compared with physicians.

The results also revealed that men had significantly higher RMRS total scores and all subscale scores compared to women. Considering previous reports that nurses have higher levels of stress and lower levels of mental health compared to physicians [25, 41, 42, 45] and that women were more likely to suffer burnout and poorer mental health during the COVID-19 pandemic [45, 46], it is possible that lower levels of moral resilience among women and nurses contributed to these outcomes, even prior to the pandemic.

The current findings indicated that technicians, pharmacists, and therapists also exhibited lower RMRS scores compared to physicians. Previous studies have reported that chaplains exhibited higher RMRS scores than physicians and nurses in response to moral adversity and nurses exhibited lower moral efficacy compared to other professions [25]. These results suggest that moral distress is commonly experienced by nonphysician healthcare providers in Japan, highlighting the need to investigate moral distress across a wider range of professions in clinical practice.

A correlation was found between higher levels of education and the total score of moral resilience. This result may reflect the current situation in Japan's healthcare system, in which physicians largely hold the authority for final decision-making regarding patient team care. In addition, individuals with advanced education in the Japanese medical field are mainly physicians or other healthcare professionals directly involved in clinical decision-making. This context may have somewhat influenced the current results, which suggested that moral resilience tended to be lower among nonphysicians, while higher education levels were associated with higher moral resilience scores. However, when examining differences across subscales, it was shown that personal integrity and moral efficacy were not necessarily correlated with higher levels of education, suggesting that elements of moral resilience are not only developed through education but also through individual attributes and experiences. Therefore, a more detailed analysis is needed regarding the attributes associated with high moral resilience.

### 4.3. Differences From the Original RMRS-16 and Cultural Background

Analysis of survey participants' responses revealed that the average overall score of the RMRS was 2.66 (SD: 0.41). This was lower than the average score of 3.04 (SD: 0.4) reported in the original survey [1]. The results shown in Table 4 indicate that the average scores in all subscales of the RMRS were lower than those in the original survey.

The differences between the overall scores in this study and the original research [1] suggest that there may be differences in understanding the concepts of morality and ethics caused by cultural differences. In some countries, morality and ethics may be understood in association with religious faith and behavior. In the cultural context of Japan, in which levels of religious consciousness are relatively low, it is likely that many healthcare professionals primarily learn about concepts of morality and ethics through education. Therefore, the low scores on the RMRS among healthcare professionals in Japan may be related to a lack of awareness or understanding regarding the actions necessary to maintain their own morality, which is a key concept in moral resilience.

Importantly, the current study revealed strong positive correlations between responses to moral adversity and relational integrity. In addition, we found strong positive correlations between personal integrity and moral efficacy. The meaning of integrity is multifaceted, encompassing competencies such as commitment to moral principles, consistency, context, continuity, and the ability to demonstrate these qualities to maintain wholeness [47, 48]. However, among healthcare professionals in Japan, there may be a lack of understanding of the multifaceted nature of the concept of integrity. Specifically, as indicated by the results of this study, it is possible that maintaining consistency with the values of others may be perceived as encountering adversity, and the concept of personal consistency may be perceived as closely related to skills such as moral resilience. These findings suggest that the concept of integrity may not be well-established among healthcare professionals in Japan.

### 4.4. Limitations of the Study and Utility of the Scale

We conducted an initial validation and reliability study of the Japanese version of the RMRS. This study provides representative data on moral resilience among healthcare workers in Japan. The current findings support the use of moral resilience as an effectiveness indicator for intervention programs aimed at mitigating and preventing moral distress and moral injury and contributing to the development of more advanced intervention programs. In addition, the data presented in this study may serve as reference data for the development of intervention programs aimed at enhancing moral resilience among nurses and healthcare workers during outbreaks of emerging infectious diseases and disasters, who are prone to burnout and moral distress.

A potential limitation of the current study is related to the reliability of the data. This study employed an online survey, which may decrease the credibility of respondents' answers, and delineating results that were specific to certain occupations or fields was challenging. Therefore, it is essential to revalidate the survey in the future by administering it offline to ensure the quality of the attributes and further enhance its reliability and validity. In addition, the current survey results suggest that there is a broad spectrum of understanding of moral-related terminology among healthcare workers in Japan, potentially indicating that measuring the concept of moral resilience consistently could be difficult. Furthermore, in this study, the evaluation of structural validity was conducted using the same methods employed in previous research. Given that this scale consists of ordinal data, the scale structure may be better explained by conducting factor analysis using the weighted least squares mean and variance adjusted or Bayesian estimation methods.

Despite the limitations described above, the results suggested some structural consistency, indicating that the scale's validity is supported by findings similar to those of previous studies conducted in other countries. In future, conducting revalidation and ensuring validity across various healthcare settings while fostering concepts related to morality among healthcare workers in Japan will further enhance the utility of the RMRS.

## 5. Conclusions

The current findings assessed the reliability and validity of the Japanese version of the RMRS. Cronbach's alpha exceeded 0.70 for all subscales. On the basis of the fit indices of the CFA, the structural validity of the Japanese version was generally supported. Criterion-related validity was also acceptable. Regarding the utility of the Japanese version of the RMRS, the findings are consistent with previous surveys of mental health among healthcare workers. These results suggest that moral resilience may be a useful indicator for measuring the mental health status of healthcare professionals. However, the ICC values for the subscales of the RMRS were below acceptable levels, and the results of the standardized residual covariances also suggested a model misfit. These results suggest that healthcare professionals in Japan may not be familiar with the concept of moral resilience, and the distinction between subscales may be ambiguous because of cultural differences. These challenges have also been reported in relation to the original [1] and Turkish versions [49] of the RMRS, indicating the need for further refinement of the concept. Moreover, considering the necessity of cross-cultural comparisons, the Japanese version of the RMRS retained the same four-factor structure as the original version, allowing for its continued use and comparison across different contexts. Therefore, conducting a conceptual analysis of moral resilience in Japanese individuals and developing scales that are more tailored to the Japanese population are important topics for future research.

## Figures and Tables

**Table 1:** Participant characteristics (*n* = 1295).

	*N* (%) or *M* (SD)
Age (years), M (SD)	46.4 (12.4)
Years of experience in current certification, M (SD)	21.1 (12.3)
Gender, *n* (%)	
Male	663 (51.2)
Female	632 (48.8)
Role, *n* (%)	
Physician	514 (39.7)
Nurse	498 (38.5)
Therapist	102 (7.9)
Pharmacologist, technician	96 (7.4)
Social worker	20 (1.5)
Others	65 (5.0)
Primary practice location, *n* (%)	
Outpatient	442 (34.1)
Inpatient—acute care, surgical	329 (25.4)
Inpatient—chronic care, mixed ward	259 (20.0)
Welfare department	41 (3.2)
Maternity department	28 (2.2)
Others	170 (13.1)
Employment role, *n* (%)	
Staff	962 (74.3)
Administration	289 (22.3)
Others	44 (3.4)
Educational background (*n* = 1287), *n* (%)	
University	574 (44.6)
Vocational colleges	439 (34.1)
Graduate school	256 (19.9)
Others	18 (1.4)

*Note:* Other roles: psychologist, clinical engineer, nutritionist, and dental hygienist; other practice locations: dialysis unit, operating room, clinical laboratory, rehabilitation department, and nutrition department; other employment roles: nonregular staff and part-time workers; missing values occurred in the educational background because participants were allowed to answer, “no response.”

Abbreviations: M, mean; SD, standard deviation.

**Table 2:** Intraclass correlation coefficients for the revised RMRS-16 in test–retest analysis (*n* = 96).

Scale	Intraclass correlation coefficient	95% confidence interval	*p* value
Response to moral adversity	0.63	0.50–0.74	< 0.001
Personal integrity	0.48	0.31–0.62	
Relational integrity	0.45	0.27–0.59	
Moral efficacy	0.53	0.37–0.66	
Total scale	0.70	0.58–0.79	

Abbreviation: RMRS, Rushton Moral Resilience Scale.

**Table 3:** Confirmatory factor analysis results.

Indexes	*χ* ^2^	df	*χ* ^2^ */*df	GFI	AGFI	CFI	SRMR	RMSEA
The Japanese version of the RMRS-16 model	697.31	98	7.115	0.935	0.909	0.917	0.055	0.069

Abbreviations: AGFI, adjusted goodness-of-fit index; CFI, comparative fit index; df, degrees of freedom; GFI, goodness-of-fit index; RMSEA, root mean square error of approximation; SRMR, standardized root mean square residual.

*p*  <  0.001.

**Table 4:** Correlation between the subscale factors of the RMRS and other measures.

	*n*	M (SD)	1. RMRS_response to moral adversity	2. RMRS_personal integrity	3. RMRS_relational integrity	4. RMRS_moral efficacy	5. RMRS_total	6. K6	7. MBI-GS	8. MMD-HP	9. JMES	10. RS
1. RMRS_response to moral adversity	1295	2.62 (0.63)										
2. RMRS_personal integrity	1295	2.80 (0.53)	0.19⁣^∗∗^									
3. RMRS_relational integrity	1295	2.53 (0.59)	0.60⁣^∗∗^	0.40⁣^∗∗^								
4. RMRS_moral efficacy	1295	2.68 (0.53)	0.13⁣^∗∗^	0.62⁣^∗∗^	0.28⁣^∗∗^							
5. RMRS_total	1295	2.66 (0.41)	0.70⁣^∗∗^	0.73⁣^∗∗^	0.80⁣^∗∗^	0.67⁣^∗∗^						
6. K6	648	2.59 (3.46)	−0.45⁣^∗∗^	−0.14⁣^∗∗^	−0.36⁣^∗∗^	−0.16⁣^∗∗^	−0.41⁣^∗∗^					
7. MBI-GS	648	54.90 (19.05)	−0.51⁣^∗∗^	−0.30⁣^∗∗^	−0.40⁣^∗∗^	−0.34⁣^∗∗^	−0.55⁣^∗∗^	0.68⁣^∗∗^				
8. MMD-HP	648	104.95 (68.02)	−0.42⁣^∗∗^	−0.04	−0.23⁣^∗∗^	−0.01	−0.27⁣^∗∗^	0.38⁣^∗∗^	0.45⁣^∗∗^			
9. J-MES	648	20.43 (8.46)	0.24⁣^∗∗^	0.50⁣^∗∗^	0.37⁣^∗∗^	0.54⁣^∗∗^	0.57⁣^∗∗^	−0.23⁣^∗∗^	−0.48⁣^∗∗^	−0.11⁣^∗∗^		
10. RS	648	49.84 (12.63)	0.37⁣^∗∗^	0.44⁣^∗∗^	0.39⁣^∗∗^	0.41⁣^∗∗^	0.57⁣^∗∗^	−0.46⁣^∗∗^	−0.59⁣^∗∗^	−0.21⁣^∗∗^	0.57⁣^∗∗^	

*Note:* Missing data occurred on the K6, MBI, MMD-HP, JMES, and RS because they were not included in the second survey that some participants opted to take.

Abbreviations: J-MES, the Japanese version of the Moral E5cacy Scale; K6, Kessler Psychological Distress Scale; MBI, the Japanese version of Maslach Burnout Inventory-General Survey; MMD-HP, the Japanese version of the Measure of Moral Distress for Healthcare Professionals; RMRS, Rushton Moral Resilience Scale; RS, the Japanese version of the Resilience Scale and short version.

^∗∗^Means *p* < 0.001.

**Table 5:** RMRS total scores and factor scores compared between the Japanese version of the RMRS in the current study and the RMRS original study [1].

	*n*	The present study (*n* = 1295)	*n*	RMRS original study	*t*-test
		M (SD)		M (SD)	
RMRS total score	1295	2.66 (0.41)	1258	3.04(0.45)	*p* < 0.001
RMRS adversity	1295	2.62 (0.63)	1253	2.77(0.68)	
RMRS personal integrity	1295	2.80 (0.53)	1257	3.30(0.52)	
RMRS relational integrity	1295	2.53 (0.59)	1257	2.96(0.65)	
RMRS moral efficacy	1295	2.68 (0.53)	1254	3.14(0.56)	

Abbreviations: M, mean; RMRS, Rushton Moral Resilience Scale; SD, standard deviation.

**Table 6:** Differences in moral resilience score by gender, professional role, practice location, and educational background.

	*n*	Response to moral adversity			Personal integrity			Relational integrity			Moral efficacy			Moral resilience total		
		M	SD	*p*	M	SD	*p*	M	SD	*p*	M	SD	*p*	M	SD	*p*
Gender	1295															
Male	663	2.77	0.64	< 0.001	2.90	0.55	< 0.001	2.69	0.60	< 0.001	2.76	0.54	< 0.001	2.78	0.43	< 0.001
Female	632	2.46	0.57		2.69	0.49		2.34	0.55		2.59	0.5		2.53	0.35	
Professional role	1295															
Physician, dentist	514	2.80^b,d,e,f^	0.63	< 0.001	2.94^b,c,d,f^	0.55	< 0.001	2.73^b,c,d,f^	0.60	< 0.001	2.80^b,c,d,f^	0.52	< 0.001	2.82^b,c,d,f^	0.43	< 0.001
Nurse	498	2.46^a,c^	0.60		2.71^a^	0.50		2.38^a^	0.56		2.59^a^	0.51		2.54^a^	0.36	
Pharmacist, technician	96	2.70^a,b,f^	0.63		2.74^a^	0.51		2.49^a^	0.54		2.59^a^	0.50		2.63^a^	0.39	
Therapist	106	2.57^a^	0.50		2.64^a^	0.47		2.48^a^	0.55		2.56^a^	0.50		2.56^a^	0.33	
Social worker	20	2.29^a^	0.70		2.84	0.58		2.61	0.65		2.90	0.58		2.66	0.50	
Oher medical worker	61	2.41^a,c^	0.61		2.60^a^	0.48		2.29^a^	0.50		2.59^a^	0.45		2.47^a^	0.36	
Employment role	1295															
Staff	962	2.58^b,c^	0.39	< 0.001	2.74^b,c^	0.52	< 0.001	2.47^b,c^	0.58	< 0.001	2.62^b^	0.52	< 0.001	2.60^b,c^	0.39	< 0.001
Administration	289	2.71^a^	0.44		2.94^a^	0.54		2.70^a^	0.61		2.84^a^	0.52		2.80^a^	0.44	
Others	44	2.86^a^	0.62		2.94^a^	0.61		2.76^a^	0.56		2.80	0.52		2.84^a^	0.42	
Primary practice location	1295															
Outpatient	442	2.71^b,c,d^	0.65	< 0.001	2.88^b,c,f^	0.51	< 0.001	2.61^b,c^	0.59	< 0.001	2.74^c^	0.50	< 0.001	2.74^b,c,d^	0.42	< 0.001
Inpatient—acute care, surgical	329	2.56^a^	0.57		2.77^a^	0.51		2.48^a^	0.56		2.66	0.50		2.62^a^	0.39	
Inpatient—chronic care, mixed ward	259	2.53^a^	0.66		2.74^a^	0.55		2.43^a^	0.60		2.61^a^	0.57		2.58^a^	0.41	
Welfare department	41	2.31^a^	0.58		2.73^a^	0.52		2.40	0.61		2.66	0.60		2.52^a^	0.39	
Maternity department	28	2.68	0.67		2.78	0.55		2.54	0.64		2.68	0.44		2.67	0.48	
Others	196	2.70	0.60		2.73	0.57		2.61	0.61		2.63	0.54		2.67	0.41	
Educational back ground	1287															
Graduate school	256	2.78^b,c,d^	0.66	< 0.001	2.99^b,d^	0.54	< 0.001	2.88^b,c,d^	0.64	< 0.001	2.85^b,c^	0.54	< 0.001	2.85^b,c,d^	0.45	< 0.001
University	574	2.63^a,c^	0.62		2.77^a^	0.55		2.51^a^	0.56		2.65^a^	0.52		2.64^a,c^	0.41	
Vocational colleges	439	2.52^a,b^	0.60		2.71	0.48		2.43^a^	0.56		2.59^a^	0.50		2.56^a,b^	0.35	
High school/others	18	2.31^a^	0.54		2.75^a^	0.48		2.26^a^	2.26		2.75	0.58		2.51^a^	0.29	

*Note:* Means with the same superscript letter were significantly different at the *p* < 0.05 level using one-way analysis of variance with Tukey's follow-up tests. Other roles: psychologist, clinical engineer, nutritionist, and dental hygienist; other employment roles: nonregular staff and part-time workers; other practice locations: dialysis unit, operating room, clinical laboratory, rehabilitation department, and nutrition department.

## Data Availability

The data used to support the findings of this study are available from the corresponding author upon request. The data are not publicly available due to privacy or ethical restrictions.
